# Conflict wounds and antimicrobial resistance: a systematic review on local antimicrobials to prevent infection after extremity open fracture

**DOI:** 10.1136/military-2026-003255

**Published:** 2026-06-02

**Authors:** Henry A Claireaux, Scott JC Pallett, Luke SP Moore, Andrew M Edwards, Julian R Jones, Matthew K O’Shea, Arul Ramasamy

**Affiliations:** 1 Department of Materials, Imperial College London - South Kensington Campus, London, England, UK; 2 Academic Department of Military Trauma and Orthopaedics, Birmingham, UK; 3 Centre for Bacterial Resistance Biology, Imperial College London - South Kensington Campus, London, England, UK; 4 Clinical Infection Department, Chelsea and Westminster Hospital NHS Foundation Trust, London, England, UK; 5 Centre of Defence Pathology, Queen Elizabeth Hospital Royal Centre for Defence Medicine, London, England, UK; 6 National Institute for Health Research Imperial Biomedical Research Centre, Imperial College London - Hammersmith Campus, London, England, UK; 7 Department of Infectious Disease, Imperial College London - South Kensington Campus, London, England, UK; 8 Centre of Defence Pathology, Queen Elizabeth Hospital Royal Centre for Defence Medicine, Birmingham, UK; 9 Department of Immunology and Immunotherapy, University of Birmingham College of Medical and Dental Sciences, Birmingham, England, UK; 10 Centre for Injury Studies, Imperial College White City Campus, London, UK

**Keywords:** ORTHOPAEDIC & TRAUMA SURGERY, Adult orthopaedics, Trauma management, ACCIDENT & EMERGENCY MEDICINE

## Abstract

**Introduction:**

Contemporary conflict-associated injuries with open fractures are challenged by infection and antimicrobial resistance (AMR), compromising empiric antimicrobials and limiting options for systemic treatment. Local antimicrobials in high concentrations may offer an adjunctive therapy but the evidence and its relevance to conflict settings are unclear.

**Methods:**

A systematic review was undertaken using the Medline, Embase and Cochrane databases, searching for evidence relating to the use of local antimicrobials to prevent infection after extremity open fracture. Preferred Reporting Items for Systematic Reviews and Meta-Analyses (PRISMA) guidelines were followed. The review considered evidence for the effect of local antimicrobials across different agents, different causes of infection, AMR and adverse effects. Available evidence was considered in the context of its relevance to conflict injuries and priority research gaps were identified.

**Results:**

No studies were identified in conflict or humanitarian settings. Seven civilian systematic reviews and meta-analyses considering different outcomes and four randomised controlled trials (RCTs) were identified. There was low-quality evidence for a reduction in infection when using prophylactic local antimicrobials in addition to systemic antimicrobials, and there was no evidence of adverse outcomes. Evidence skewed towards use for Gram-positive infections. There was a lack of level one evidence and marked heterogeneity across study designs.

**Conclusion:**

AMR has emerged as a major challenge for the management of open fractures sustained in conflict. There is an urgent requirement to identify alternative treatment options and adjuncts. Local antimicrobials and biomaterials present potential opportunities. Further evidence from RCTs is required across all settings. In the context of conflict, this must include relevant outcomes, including the impact of delayed surgery, wound contamination and AMR.

**WHAT IS ALREADY KNOWN ON THIS TOPIC**

**WHAT THIS STUDY ADDS**

- This study highlights the urgent need for high-quality evidence on early local antimicrobial use to prevent open fracture-related infection in conflict, guiding future research agendas and study design.

**HOW THIS STUDY MIGHT AFFECT RESEARCH, PRACTICE OR POLICY**

- Findings support reconsideration of current military infection-prevention practice by identifying local antimicrobials as a plausible adjunct where the effectiveness of systemic prophylaxis is limited by AMR.
- The work highlights an opportunity to improve preparedness for LSCO and the need for pre-approved, prospective research protocols across allied militaries.

## Introduction

 Contemporary large-scale combat operations (LSCO) confer a selection pressure that drives the emergence and dissemination of antimicrobial resistance (AMR).^1^ In recent years, there has seen a considerable increase in the use of unmanned drone technology and ballistics,^2^ with resultant injuries involving the transfer of huge energy to tissue, along with significant environmental contamination.^3^ Non-viable tissue and foreign material then become a nidus for infection.

Evacuation for surgical wound excision (debridement) may be delayed by several days in the contemporary operating environment,^4^ increasing reliance on broad-spectrum prophylactic antimicrobials. In contrast to recent UK experiences during conflicts in Iraq and Afghanistan, the medical evacuation chain in the current LSCO typically involves passage through multiple healthcare facilities where overcrowding limits capacity for infection prevention and control and increases the likelihood of acquiring resistant bacteria.^5^ In addition, microbiology diagnostic capabilities may be limited,^6^ with patients moving along the evacuation chain before culture results are available to guide the use of targeted or narrow-spectrum antimicrobial therapy.

Extensive AMR has been identified among combat casualties in the Russo-Ukraine war, particularly for infections involving Gram-negative bacteria.^7^ Surveillance data have been very limited, but studies show high rates of resistance to reserve antimicrobials (including over 80% resistance rates for meropenem), emergence of unusual patterns of resistance and occasions of pan-resistant infections for which no commercially available antimicrobial options remain.^7–10^ Multidrug-resistant (MDR) wound infections have been identified in patients early (within a week) after injury and at initial surgery.^7^ Ukrainian antimicrobial guidelines recommend cefazolin or, where there is expectation of delayed evacuation, first-line choice as prophylaxis against infection after traumatic injury is an oral fluoroquinolone (moxifloxacin or levofloxacin) or intravenous ertapenem.^11^ However, high rates of quinolone resistance have been identified in this conflict.^7 12^ UK military guidelines recommend co-amoxiclav for post-trauma prophylaxis,^13^ but bacteria isolated from wounded patients in this conflict are frequently resistant to beta-lactam and beta-lactam/lactamase-inhibitor combination antimicrobial agents (including co-amoxiclav).^7–10 12^ Data are likewise limited across other conflict areas, where conditions that promote AMR are similar, and this issue is unlikely to be restricted to Ukraine. Therefore, AMR is a major threat to health within conflict zones and to wider healthcare systems as resistance spreads regionally through displaced populations, returning travellers, or evacuation for further surgical management.^8 10 14^

Removing contamination and excising devitalised tissue greatly reduces the chance of infection and resulting morbidity and mortality for patients with open fractures sustained in conflict. Around 20% of open tibial fractures are complicated by infection across civilian and military studies.^15–17^ In a UK military cohort of patients with blast injuries to the foot and ankle, those in whom limb salvage was attempted had an infection rate of 42% and an osteomyelitis rate of 12%.^18^ In the conflicts of Iraq and Afghanistan, rapid evacuation for resuscitation and wound excision was deliverable within hours. Unfortunately, such rapid surgical treatment is unlikely to be achievable in a peer-on-peer or near-peer conflict and, given the emergence of high rates of resistance to systemic antimicrobials, alternative approaches are urgently required. Selection of systemic antimicrobials is based on assessment of minimum inhibitory concentrations (MICs). MICs represent the lowest concentration required to inhibit visible growth of an organism and are interpreted using clinical breakpoints set by international scientific advisory committees, taking into consideration experimental data, clinical outcomes, pharmacokinetics and pharmacodynamics of the drug in question.^19^ While dosing options for systemic antimicrobials can be limited by dose-related side effects, local antimicrobials applied directly to wounds can deliver concentrations several orders of magnitude higher than the MIC without the same risk of systemic toxicity.^20^ Local antimicrobials have been used as an adjunct to the treatment of osteomyelitis and prosthetic joint infection for decades and can be delivered in several ways.^21^ Most simply, powdered antimicrobial can be applied to the area where it is rapidly absorbed, resulting in a short duration of action. Alternatively, antimicrobials can be delivered on a carrier such as polymethylmethacrylate cement or calcium sulphate/phosphate, which aims to provide sustained high concentrations of antimicrobial over time. Commercially available preparations frequently include gentamicin, clindamycin, vancomycin, tobramycin, or rifampicin.^22^ Because delivery is direct, the problems of poor blood supply to bone and limited antimicrobial penetrance of bone are minimised. In this way, local antimicrobials may offer an adjunctive early management option for suspected or confirmed open fracture conflict-associated infections pending access to definitive surgery, but their potential in this setting is less well understood.

We undertook a systematic review of the current evidence for the use of local antimicrobials in preventing open fracture-related infection and considered its relevance for conflict settings, identifying gaps and opportunities for priority actions and future research in this area.

## Methods

To understand the current evidence base for use of local antimicrobials to prevent infection after extremity open fractures and its relevance in a conflict setting, an initial search for level one evidence was conducted on 25 October 2025 across Medline, Embase and Cochrane databases using broad search terms including “local OR topical OR intrawound” AND “antimicrobial OR antimicrobial*” AND “fracture*” AND “random* OR RCT OR review OR meta-anal*”. Preferred Reporting Items for Systematic Reviews and Meta-Analyses (PRISMA) guidelines were followed (figure 1). To review the most relevant evidence for conflict injuries, inclusion criteria were any randomised controlled trial (RCT), systematic review or meta-analyses that considered local antimicrobial use in the management of extremity open fractures in adults >18 years old and where the primary outcome was infection. Studies conducted in conflict, humanitarian and civilian settings were eligible for inclusion. This is because it is challenging to perform research during conflict and natural disasters, meaning fewer papers tend to exist. Furthermore, civilian research may be generalisable to conflict, with caveats. Reference lists of included studies were checked for additional relevant manuscripts. Exclusion criteria included individual case reports, small case series or studies or any study reporting on local antimicrobial use for non-extremity (eg, spinal) or closed fractures or where primary outcomes were other than assessment of fracture-related infections. In the context of military operations, X-ray is not available to assess high-energy (blast and ballistic) extremity injuries presenting to a combat medical technician or general practitioner far forward in the battlespace (role one). Therefore, clinical decisions are made on the assumption that high-energy wounds may represent open fractures.

**Figure 1:**
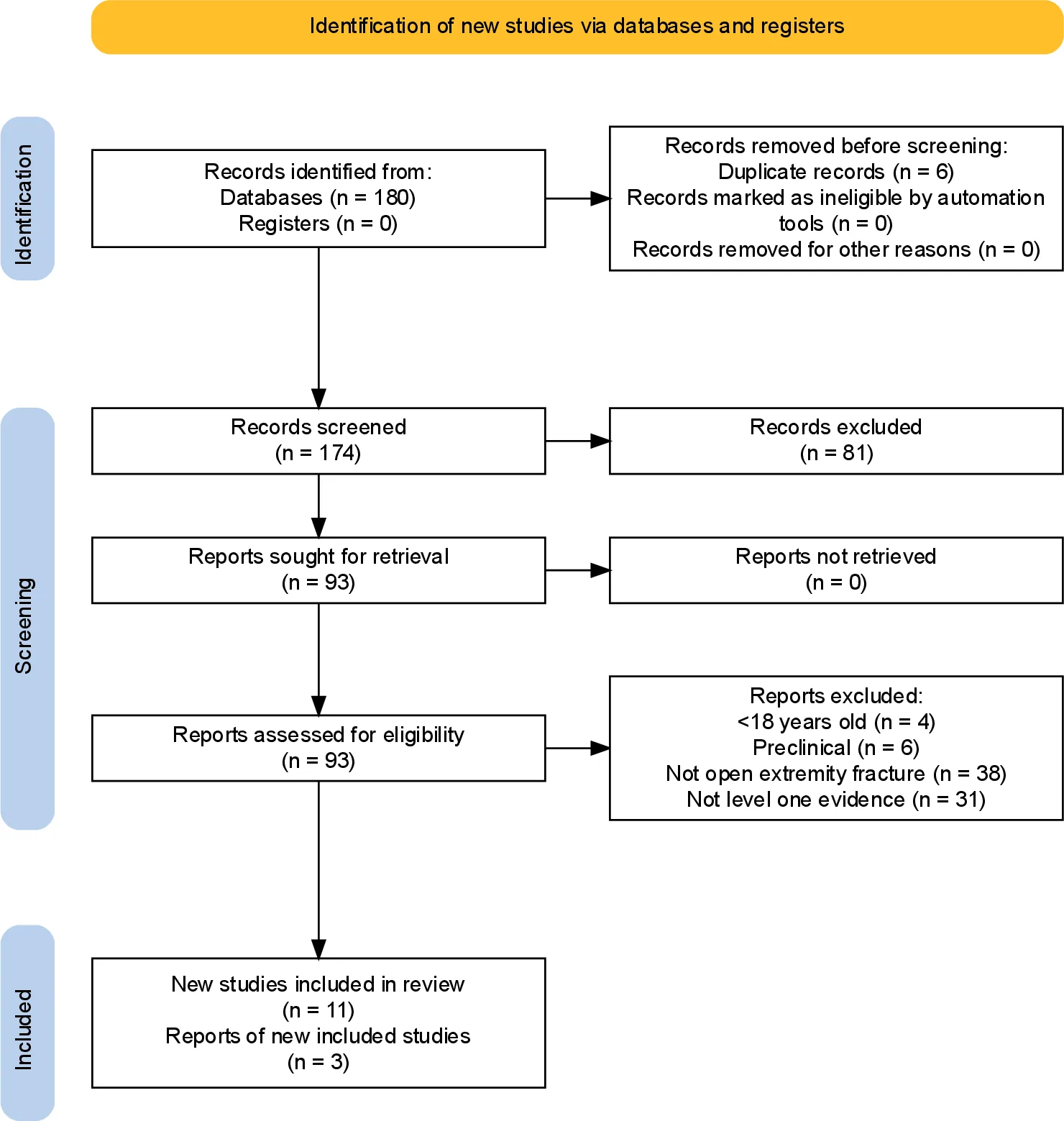
Preferred Reporting Items for Systematic Reviews and Meta-Analyses flow chart of included studies.

Identified studies were screened independently by two of the authors (HAC and SJCP) for the relevance of available research to conflict settings/injury patterns; evidence of prophylactic effect on preventing infections in open limb fractures; evidence for different antimicrobial agent and pathogen combinations and evidence for associated adverse effects. Data were extracted independently into a bespoke spreadsheet. The collated evidence was subsequently considered in the context of the ongoing conflict in Ukraine and key research gaps, and priorities were identified. The certainty of evidence was assessed using the Grading of Recommendations Assessment, Development and Evaluation (GRADE) approach.^23^

## Results

The literature search identified 180 unique manuscripts. No studies on local antimicrobial use to prevent infection after open fracture sustained in conflict or humanitarian settings were identified. In the civilian literature, however, seven systematic reviews and meta-analyses were identified.^21 24–29^ Reviews considered adverse events, including increasing Gram-negative infections (by using local vancomycin), renal injury and non-union.^24 26 27 29^ Four civilian RCTs were identified (table 1).^30–33^ The included systematic reviews summarised 13 observational studies at considerable risk of bias, of which 11 were retrospective and two were prospective in design.^21 24–29^ Three ongoing, registered RCTs were also identified (table 1).^34–36^ Finally, four sub-studies were identified, nested within cited RCTs with a focus on potential side effects (eg, AMR pressures, renal toxicity).^37–40^ The certainty of evidence (GRADE approach) is very low because no conflict or humanitarian studies were identified. Evidence is, therefore, extrapolated from civilian populations and downgraded for indirectness, inconsistency, and imprecision.^23^

**Table 1:** Study features of randomised controlled trials considering the use of local antimicrobials during the management of open extremity fractures, where the primary outcome is prevention of infection

Study	Injury patterns	n	Antimicrobial(s)	Dose, preparation, frequency	Comparator	Primary outcome follow-up	Primary outcome result	Adverse effects
Major Extremity Trauma Research Consortium, (TOBRA)^33^	LL	1528	Vancomycin and tobramycin	1 g Vanc, 1.2 g Tobra. Powder. Once.	Vancomycin powder+systemic antimicrobials	182 days	↑Deep SSI HR 2.08	NS
O’Toole *et al,* (VANCO)^30^	LL	LA=481 Control=499	Vancomycin	1 g. Powder. Once.	Systemic antimicrobials only	12 months	↓GPC infection, p*=*0.02	NU, SSSI, WD
Seenappa *et al*,^31^	UL, LL	LA=32 Control=32	Vancomycin	2 g. PMMA beads. Once.	Systemic antimicrobials only	6 months	↓FRI, NS, p*<*0.072	–
Moehring *et al,*^32^	UL, LL	LA+SA=12 LA only=22 Control=33	Tobramycin	2.4 g. PMMA beads. Once.	Systemic antimicrobials only	Mean 15 months	NS	NU
Aneja *et al*,^34^	UL, LL	600	Tobramycin	80 mg. Aqueous. At each debridement.	Systemic antimicrobials only	12 months	Pending	NU, AKI
Wheeler *et al*, (POWDER)^35^	UL, LL	200	Vancomycin	2 g. Powder. Once.	Systemic antimicrobials only	12 months	Pending	SSSI
Haonga *et al,* (GO-Tibia)^36^	LL	890	Gentamicin	80 mg. Aqueous. Once.	Systemic antimicrobials+saline	12 months	Pending	NU

All three pending RCTs are currently recruiting. Of adverse events listed, no significant association with local antimicrobial use was identified.

FRI, fracture associated infection; GPC, Gram positive cocci; LL, lower limb; NS, non-significant; NU, non-union; PMMA, polymethylmethacrylate; SSSI, superficial surgical site infection; UL, upper limb; WD, wound dehiscence.

### Study settings

In all cases, evidence summaries were formed from RCTs or observational studies conducted in sterile surgical theatre settings of predominantly high-income countries, limiting the direct relevance of findings to the resource-limited settings of conflict or humanitarian crisis. A pilot, blinded RCT considering the impact of local gentamicin solution in the management of open tibia fractures in Tanzania was also identified, demonstrating feasibility for conducting trials in low-middle income countries, but outcome data from the larger trial are still pending.^36^ Two RCTs are underway that aim to recruit injuries sustained from mixed military and civilian populations.^34 35^ Of note, one study applied local vancomycin and tobramycin to open lower extremity fractures in the emergency department, finding a non-significant reduction in subsequent cultured infections (9.1% vs 17.1%, p*=*0.13),^41^ although the study was likely underpowered (n=66 intervention, 129 controls).

### Antimicrobial use and diagnostic microbiology in local antimicrobial studies

There was marked heterogeneity across studies in antimicrobial agent choice, delivery mechanism, dose and comparator. Pooled analysis of results from included studies was, therefore, precluded. Six studies assessed local use of tobramycin (of which 4/5 studies were >25 years old, when patterns of AMR were very different to today), six of vancomycin, four of vancomycin and/or tobramycin, and in one, it was left to the prescribers to decide, with vancomycin and gentamicin being most commonly chosen.^19–27^ There remains no standardisation for the delivery or monitoring of local antimicrobial use, limiting comparison across studies.

Detailed diagnostic microbiology was often lacking, with unclear criteria used to determine positive culture significance, and infections were often described simply as “Gram-positive”, “Gram-negative” or “polymicrobial”. Some studies excluded those with polymicrobial results, and some counted polymicrobial Gram-negative infections as a single event. Where identified, *Staphylococci* were most often reported, with observations for AMR limited to classification of methicillin-resistant *S. aureus*, or unclear definitions/mechanisms.

### Assessment of local antimicrobial effect on infection rates

There was also marked heterogeneity among studies of injury mechanism and severity, definition of deep versus superficial wounds, injury site and timing of local antimicrobial application. Overall, systematic reviews concluded that evidence favoured a reduction in infections where local antimicrobials were used over systemic antimicrobials alone.^24–29^ In general, reviews commented on a likely reduction in Gram-positive infection rates, with several concluding significant reductions in incidence of infection when local antimicrobials were used (for deep wound infection risk in fracture fixation OR=0.58, 95% CI 0.39 to 0.86, p=0.007;^28^ and (majority lower limb) open fracture repair OR=0.49, 95% CI 0.37 to 0.65;^29^ OR=0.50;^26^ 95% CI 0.34 to 0.72, p<0.001 or incidence of 7.3% vs 10.1%, p=0.02).^27^ Others made more tempered conclusions (eg, a non-significant 23% reduction in deep wound infections).^24^ Authors were also clear to highlight a lack of robust level one data, however, limiting generalisability and hampered by small sample sizes, heterogeneous study design, interventions and outcomes.^27^ Conclusions were strongly affected by the one included RCT using vancomycin,^30^ with additional identified RCTs finding that topical administration of vancomycin did not reduce surgical site infections, though the incidence was low in both groups and sample sizes were small (table 1).^31 32^ The 2026 TOBRA RCT showed that the combination of intrawound tobramycin and vancomycin powder at the time of definitive fixation was associated with more deep surgical site infections than when vancomycin powder was given alone in the open fracture subgroup (HR 2.08, 95% credible interval 1.06 to 4.37) (table 1).^33^

Focus and evidence appeared stronger for the effect on reducing Gram-positive infections, although this may have been strongly linked to the 2021 RCT using vancomycin powder. There was less evidence available for the effect on reducing Gram-negative infections, where assessment tended to focus on rates as a potential adverse effect of vancomycin treatment rather than a positive outcome of its own (likely due to greater focus on vancomycin activity among larger studies), and for which one review concluded there was no available evidence.^29^ *Acinetobacter* spp. and *Klebsiella* spp. infections were very infrequently represented.

### Adverse events associated with local antimicrobial use

There were insufficient sample sizes to draw firm conclusions on adverse effects and potential effects on AMR. Local use of vancomycin has been hypothesised as a risk for increasing incidence of Gram-negative and/or resistant infections. Reviews tended to conclude no evidence for an effect on Gram-negative infection rates, while nested-RCT reporting concluded “*cautious reassurance*” that local antimicrobial use did not lead to increased resistance.^27 39 40^ Where non-union rates were commented on,^24 26 27 29^ no significant differences were found in those that received local antimicrobials, nor was there evidence of significant effects on renal function,^24 26 27^ although studies were likely underpowered. A further recent study on local tobramycin powder use likewise found no evidence for toxic serum levels or long-term changes to renal function,^37^ while another reported no significant effects following cumulative exposure to local vancomycin.^38^ Further studies looking specifically at non-union have likewise found no difference in rates when topical antimicrobials are used, but larger prospective studies are required to confirm findings.^42^

## Discussion

Available evidence suggests that local antimicrobials may prevent infection after open extremity fracture, but there is a paucity of level one evidence. Likewise, there is a lack of evidence to suggest adverse effects on infection rates, non-union, AMR or renal function following local antimicrobial use when used in combination with systemic treatment. Significant heterogeneity in study design and small sample size precluded pooled analysis and limited any strong conclusions.

When considering the current military context, the available evidence is further limited on two key fronts. First, almost all the available evidence is from high-income countries, in operating theatres with ready access to early surgical intervention following traumatic injury. This is in stark contrast to the current conflict settings, where there may be a significant delay to definitive surgery for high-energy, heavily contaminated injuries.^5 6^ Second, much of the available evidence is skewed towards Gram-positive infections and *S. aureus,* in particular. However, conflict-associated wound infections in Ukraine are challenged by a high burden of Gram-negative (including polymicrobial infection with both lactose-fermenting and non-fermenting) organisms.^7–10 12^ In particular, infections with hypervirulent *Klebsiella pneumoniae* are a major challenge,^10^ for which there is no evidence in the current literature. It should be noted that while Vancomycin showed positive results in preventing civilian open fracture-related Gram-positive infections, this antimicrobial drug is not effective against Gram-negative bacteria.^30^

Furthermore, the collected studies assessed outcomes from operations undertaken between 1990 and 2025. The prevalence and nature of AMR have developed extensively in this time, with the greatest burden experienced in low and middle-income countries.^43^ No study was identified that reported outcomes for infections with a high level of Gram-negative AMR. Most considered effect alongside systemic antimicrobials, but reports from Ukraine show extensive AMR of Gram-negative wound infections,^7–10 12^ substantially limiting (or in some circumstances eliminating) systemic antimicrobial options. Similarly, there was limited reporting on the effect on infections associated with biofilm formation, such as *Pseudomonas aeruginosa* or hyper-capsulated *Klebsiella* spp. Here, local antimicrobials at high concentration may add considerable value in enabling activity against early infection for pathogens otherwise considered highly resistant, particularly where evacuation timelines are extended. However, outcomes will also rely heavily on early diagnostic capability, systemic antimicrobial availability and access to definitive surgery. Current data provide a very limited understanding of local antimicrobials’ potential in this setting.

Conflict-associated injuries are increasingly high-energy blast injuries resulting in limb fracture, amputation and heavily contaminated wounds.^2 3^ Wound infections associated with AMR remain a constant challenge, drastically limiting options for systemic antimicrobials and increasing reliance on agents with poorer efficacy and/or a more substantial side effect profile (eg, polymyxin B).^7–10^ The AMR challenge in this setting is likely to persist and progress in the near future, and alternative options to mitigate early infection are urgently required.

In the first instance, local antimicrobial agents and delivery devices must reflect the needs of conflict settings and associated injury patterns. UK Defence is currently conducting research in several key areas to address the challenges outlined here. First, in vitro experiments are being conducted to assess the effectiveness of locally delivered antimicrobials on PerOssal beads (OSARTIS, Germany) against MDR bacterial isolates typical of infections in the Russo-Ukraine War.^22^ Local antimicrobial delivery may improve patient outcomes by reducing the impact of infection immediately after injury, during prolonged field care, and between re-look operations prior to definitive soft tissue closure/coverage. Second, research is ongoing to phenotype and explore the AMR characteristics of current conflict-associated wound infections, to better understand evolving patterns of infection and inform the development of rapid diagnostics. This includes the use of deep RNA sequencing and using both in vitro models and ex vivo clinical samples to better understand how pathogens respond following exposure to different antimicrobial combinations. Thirdly, novel biomaterials with antimicrobial effects independent of antimicrobial medicines are being studied. S53P4 bioactive glass is an established treatment for osteomyelitis and infected non-union.^44^ S53P4 wool dressings with high surface area and good handling properties may prevent infection and promote wound healing, providing antimicrobial-free and AMR-independent options for use in conflict. Haemostatic materials, such as chitosan, could be incorporated to prevent bleeding and infection in one dressing.

Local antimicrobial delivery for open fractures sustained in conflict may reduce early bacterial burden at the point of wounding, during prolonged field care and between re-look operations. Currently, there is no level one evidence from conflict or humanitarian settings. This uncertainty should not delay implementation but rather mandate a structured evaluation to generate a clinical evidence base. Priority should be given to embedding prospective, pre-approved clinical studies and surveillance into military care pathways with standardised protocols, clinical and microbiological endpoints, and monitoring of AMR. This must consider the effectiveness of early local antimicrobial use in the context of access to surgery, polymicrobial contaminated wounds, multiple injuries, timing and type of surgery (primary vs revision, initial repair vs definitive), and AMR/systemic antimicrobial choice. Without this, the current evidence gap will persist despite ongoing conflict.

## Conclusion

AMR has emerged as a major challenge for the management of open fractures sustained in conflict. There is an urgent requirement to identify alternative treatment options and adjuncts. Local antimicrobials present a potential opportunity for which available evidence suggests a low risk of associated adverse events. Further evidence from RCTs is required across all settings and in the context of conflict, this must include relevant outcomes, including the impact of delayed surgery, wound contamination, and AMR.

## Data Availability

Data sharing not applicable as no datasets generated and/or analysed for this study.
